# Epidemiology of Indigenous Dengue Cases in Zhejiang Province, Southeast China

**DOI:** 10.3389/fpubh.2022.857911

**Published:** 2022-04-14

**Authors:** Jiangping Ren, Zhiping Chen, Feng Ling, Yangmei Huang, Zhenyu Gong, Ying Liu, Zhiyuan Mao, Chunping Lin, Hao Yan, Xuguang Shi, Rong Zhang, Song Guo, Enfu Chen, Zhen Wang, Jimin Sun

**Affiliations:** ^1^Zhejiang Provincial Center for Disease Control and Prevention, Hangzhou, China; ^2^Key Laboratory of Vaccine, Prevention and Control of Infectious Disease of Zhejiang Province, Hangzhou, China; ^3^Zhejiang Provincial Station of Emerging Infectious Disease Control and Prevention, Chinese Academy of Medical Sciences, Hangzhou, China; ^4^Hangzhou Municipal Center for Disease Control and Prevention, Hangzhou, China; ^5^Department of Tropical Medicine, Tulane School of Public Health and Tropical Medicine, Tulane University, New Orleans, LA, United States; ^6^The Center for Disease Control and Prevention of Huangyan District, Taizhou, China

**Keywords:** dengue, China, epidemiology, emergence, outbreak

## Abstract

**Objective:**

Autochthonous transmission of the dengue virus (DENV) occurred each year from 2014 to 2018 in Zhejiang province, and became an emerging public health problem. We characterized the autochthonous transmission of the DENV and traced the source of infection for further control and prevention of dengue.

**Methods:**

Descriptive and spatiotemporal cluster analyses were conducted to characterize the epidemiology of autochthonous transmission of the DENV. Molecular epidemiology was used to identify the infection source.

**Results:**

In total, 1,654 indigenous cases and 12 outbreaks, with no deaths, were reported during 2004–2018. Before 2017, all outbreaks occurred in suburban areas. During 2017–2018, five out of eight outbreaks occurred in urban areas. The median duration of outbreaks (28 days) in 2017–2018 was shortened significantly (*P* = 0.028) in comparison with that in 2004–2016 (71 days). The median onset-visiting time, visiting-confirmation time, and onset-confirmation time was 1, 3, and 4 days, respectively. The DENV serotypes responsible for autochthonous transmission in Zhejiang Province were DENV 1, DENV 2, and DENV 3, with DENV 1 being the most frequently reported. Southeast Asia was the predominant source of indigenous infection.

**Conclusions:**

Zhejiang Province witnessed an increase in the frequency, incidence, and geographic expansion of indigenous Dengue cases in recent years. The more developed coastal and central region of Zhejiang Province was impacted the most.

## Introduction

Dengue (also known as “Dengue fever”) is one of the most extensively distributed and highly concerning vector-borne diseases. This disease is spreading from tropical and subtropical to temperate regions and causing tremendous health, economic, social, and political burdens, especially in developing countries and previous unaffected regions (1). About half of the world population is now at risk of dengue (2). Globally from 1990 to 2019, the number of incident cases, deaths, and age-standardized disability-adjusted life years (DALYs) due to dengue increased gradually (3), although fluctuations have been reported in some countries (4, 5). Age-standardized incidence, death, and DALY rates accelerated most among high-middle- and high-sociodemographic-index regions. By district, South-East Asia and South Asia had most dengue-incident cases, deaths, and DALYs, East Asia had the fastest rise in age-standardized incidence rate, whereas tropical Latin America led in age-standardized death rate and age-standardized DALYs rate (3). Dengue was listed as one of 10 threats to global health in 2019 and one of neglected tropical diseases by the World Health Organization (6, 7). The first autochthonous DENV transmission since the founding of the People's Republic of China in 1949 was reported in Foshan, Guangdong Province, in 1978. Indigenous cases were reported every few years after that. From 2006, autochthonous DENV transmission was reported each year, with an increasing trend in incidence and expanding trend in geographical distribution (from southern coastal tropical or subtropical regions to southwestern regions bordering Laos and Vietnam, reaching as far as the central temperate region of China) (8). The highest dengue incidence on record in China was reported in 2014, with >40,000 cases reported, and the largest-scale dengue epidemic was reported in 2019, with 13 provinces and >72 prefecture cities affected (8).

The Dengue virus (DENV) is a positive single-stranded RNA virus belonging to the Flaviviridae family (9). Four antigenically distinct (but immunologically cross-reactive) serotypes, DENV 1 to DENV 4, have been identified (9). Each serotype is classified further into several genotypes (9). The clinical spectrum ranges from asymptomatic infection to severe dengue fever. There is no specific antiviral treatment for dengue. The fatality rates of severe dengue is <1% when early detection and proper medical care are available (2). But in some Asian and Latin American countries, severe dengue is a leading cause of serious illness and death (2). *Aedes aegypti* (followed by *Aaedes albopictus*) is the primary vector of the DENV. In mainland China, *A. albopictus* has been found throughout tropical, subtropical, and temperate zones, spanning most of the area from Hainan Province to Liaoning Province, and is the dominant mosquito species in residential areas. *A. aegypti* is found only in the coastal areas of China, within 22° north latitude, and is confined mainly to Hainan Province (10). The first licensed dengue vaccine, CYD-TDV (Dengvaxia^®^), has been used in several countries. As it increases the risk of severe dengue in seronegative vaccine recipients, individuals without history of wild DENV infection should not be vaccinated (11). And for the country considering vaccination as part of the dengue control programme, pre-vaccination screening should be conducted to exclude the seronegative people or the recent documentation of seroprevalence rates should be ≥80% by age 9 years if the screening is not feasible (11). As the low seroprevalence rates, dengue vaccine has not been licensed in China. Vector control, surveillance and health education are the three most important measures for the dengue control in China.

Zhejiang Province (ZP) is located in the southeast coast of China, and has a subtropical monsoon climate. *A. aegypti* has not been founded in ZP. However, there have been frequent reports of imported dengue cases caused by the high mobility of humans and high vector competence during later-spring, summer and early-fall in ZP. Hence, ZP has been defined as a Province carring a high-risk of a dengue epidemic by the Chinese Center for Disease Control and Prevention. The first autochthonous DENV transmission in ZP since 1949 was reported in Ningbo in 2004. With frequently report of dengue case (including imported and indigenous) and its impact on health, economic and social, this disease attracts more and more attention of the public and government in the past years in ZP. In this retrospective study, we aimed to characterize autochthonous DENV transmission, uncover the change of the epidemiology, and trace the source of infection in ZP during 2004–2018. We also aimed to provide a scientific basic of the policymaking, resource allocation, and interventions planning for the control and prevention of dengue.

## Materials and Methods

### Ethical Statement

Ethical approval of our study protocol was obtained from the Ethics Committee of the Chinese Center for Disease Control and Prevention (201214). Written informed consent was waived according to the Contagious Diseases Act of the People's Republic of China.

### Data Collection

All patients who met with the diagnostic criteria and principles of management for dengue (WS 216-2001, 2004–2007) (12), diagnostic criteria for dengue (WS 216-2008, January 2008–July 2018) (13), or diagnostic criteria for dengue (WS 216-2018, after August 2018) (14) had to be reported through the National Notifiable Disease Surveillance System within 24 h after the diagnosis, as required by the Law of the People's Republic of China on Prevention and Treatment of Infectious Diseases. Whereafter, a detailed epidemiological survey was conducted by local officials from the Center for Disease Control and Prevention. The epidemiological survey covered general individual information, progression of disease and treatment, symptoms, physical examination, laboratory tests, and contact-and-visiting history. An “indigenous case” was defined as a case who had not traveled to a dengue-endemic region within 14 days before disease onset. A dengue outbreak was identified if there were ≥3 epidemiologically related indigenous dengue cases within 14 days. The annual county-level population was downloaded from the provincial bureau of statistics of ZP (http://tjj.zj.gov.cn/).

All the sequences in this study were collected from the National Center for Biotechnology Information (NCBI; www.ncbi.nlm.nih.gov/). “Dengue” and “Zhejiang” were the keywords employed to search all potential sequences. Sequences that did not cover the viral envelope (E) gene or the sequence of the partial E gene were excluded. Only the sequences isolated from indigenous dengue cases of ZP were included in analyses. Reference sequences for the E gene of different DENV genotypes were downloaded from NCBI. Meteorological data (temperature, relative humidity, precipitation) were collected from the national meteorological data-sharing system (http://data.cma.cn/).

### Statistical Analyses

Categorical data are described as percentages. Continuous data are described as the median with interquartile range (IQR), or mean ± standard deviation (SD). Continuous data were compared using the Student's *t*-test or Wilcoxon rank-sum test. The chi-square or Fisher's exact test were used for the comparison of categorical data. The null hypothesis would be rejected if the *P* < 0.05.

SaTScan™ vesion 9.1.1 was employed to scan the space-time clusters of dengue (15, 16). The space-time cluster indicated a geographical area with more cases of a disease across a certain period than it would be expected if the risk was evenly distributed. Cylindrical scan windows with circular base and discrete Poisson models were used to identify the high-risk areas based on monthly units. The circular base indicated the scanned space and the height of the cylinder reflected the time period of potential clusters. The maximum spatial cluster size for spatial window was 50 percent of population at risk. The maximum temporal cluster size for temporal window was 50 percent of study period. Likelihood ratio tests and the log-likelihood ratios (LLR) were used to determine the significance of identified clusters and the maximum number of Monte Carlo replication was 999. Likelihood ratio tests is developed by Vuong QH for model selection and non-nested hypotheses in 1989 (17). The function was:


$$\begin{array}{l} LLR=\log{\left(\frac{c}{E\left[c\right]}\right)}^{c}{\left[\frac{C-c}{C-E\left[c\right]}\right]}^{C-c}I\left(\right) \end{array}$$

where C is the total number of cases, c is the observed number of cases within the window, and E[c] is the expected number of cases within the window under the null-hypothesis. I() is an indicator function. As SaTScan was set to scan clusters with high rates in this study, I() is equal to 1 when the window has more cases than expected under the null-hypothesis, and 0 otherwise. The null hypothesis of a spatiotemporally random distribution was rejected if the *P* < 0.05. The “window with the maximum likelihood” was defined as the primary cluster. Other a cluster with a significant LLR was defined as the “secondary potential cluster”. A map of ZP was downloaded from National Earth System Science Data Sharing Infrastructure (www.geodata.cn/). A map showing the distribution of Dengue was created by ArcGIS 10.3 (www.esri.com/).

### Phylogeny and Source Tracing

MAFFT is a multiple sequence alignment program (www.ebi.ac.uk/Tools/msa/mafft/) (18). The nucleotide sequences of E gene were aligned with MAFFT 6.843 with L-INS-i. In 1993, the first version of molecular evolutionary genetics analysis (MEGA) software (www.megasoftware.net/) was developed. It is for nucleic acid and protein sequence analysis among evolutionary and molecular biologists (19). Phylogenetic analysis was undertaken using the maximum-likelihood method. Maximum-likelihood method is one of the most widely used methods of statistical estimation (20). It was firstly added in MEGA in 2011 and is one of the most common method used to estimate phylogenetic trees now (19). A rectangular evolutionary tree was established in MEGA 7.0.25. Forty-five reference sequences of the E gene for DENV 1, 32 for DENV 2, and 19 for DENV 3 were included, respectively, to establish a rectangular evolutionary tree. Basic Local Alignment Search Tool (BLAST; https://blast.ncbi.nlm.nih.gov/Blast.cgi), with epidemiological investigation, was used to identify the most likely source of infection for autochthonous transmission.

### Vector Surveillance

Only two counties, Yiwu and Jiande, conducted surveillance for *Aedes* larval in ZP during 2004–2007. From 2008, 11 counties were selected to conduct the surveillance during June to October. In 2015, the number of counties undertaking surveillance increased to 15, and the surveillance period was May to October. From 2016, all counties in ZP were requested to conduct surveillance during April to October. The Breteau Index (BI; the number of positive containers per 100 houses) was used to indicate the density of *Aedes* larvae. Data during 2004–2007 were unavailable in this study.

## Results

### Temporal Distribution

**Figure 1:**
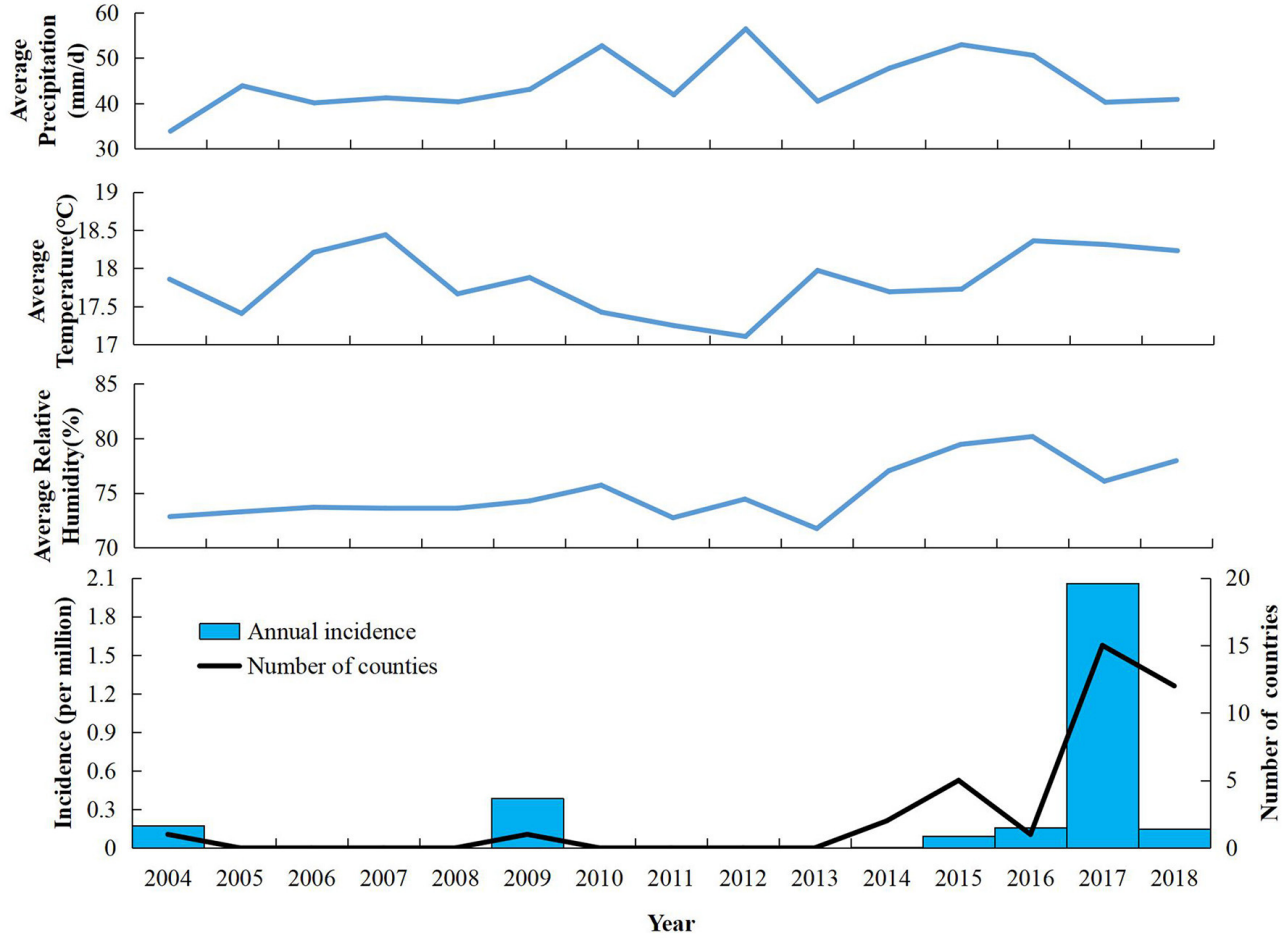
Annual incidence, number of indigenous dengue cases, and meteorology in Zhejiang Province from 2004 to 2018.

Twelve outbreaks and 1,654 cases of indigenous dengue, with no deaths, were reported in ZP during 2004–2018. In ZP, an indigenous case was not reported during 1949–2003, 2005–2008, or 2010–2013 (Figure 1). The annual mean incidence during 2004–2018 was 2.09 per million, with the two highest annual incidences reported in 2017 (20.57 per million) and 2009 (3.85 per million). Indigenous cases were reported from July to November, with the highest incidence in September (which accounted for 58.77% of the total number of cases). The monthly distributions was significantly different for 2004–2016 and 2017–2018 (χ^2^ = 150.92, *P* < 0.001). Most cases during 2004–2016 occurred in September and October, whereas a seasonal peak was noted in August and September during 2017–2018 (Table 1). The overall median duration of the outbreak was 50 (IQR, 11.75–76.25) days (Table 2). A total of 1,150 indigenous cases were reported in 2017, and 98.78% of cases were infected in Hangzhou. Autochthonous transmission of DENV in Hangzhou in 2017 was the first outbreak to occur in an urban area. All outbreaks identified in 2004–2016 were in suburban areas. However, five out of eight outbreaks during 2017–2018 occurred in an urban area. The median duration of the outbreak in 2017–2018 was significantly shorter than that in 2004–2016 (23.5 vs. 71 days, *Z* = −2.20, *P* = 0.028).

**Table 1:** Characteristics of indigenous dengue cases in Zhejiang Province from 2004 to 2018.

**Characteristic**	**2004–2016**	**2017–2018**	***P***
**Age, year (mean** **±SD)**	45.97 ± 18.972	49.72 ± 18.05	<0.001
**Male:female**	0.71:1	1.016:1	0.002
**Occupation, number (%)**			<0.001
Retiree	6 (1.78%)	366 (29.66%)	
Businessman	17 (4.14%)	170 (13.78%)	
Housework or unemployment	39 (11.54%)	162 (13.13%)	
Worker	48 (14.20%)	155 (12.56%)	
Cadre or clerk	4 (1.18%)	142 (11.51%)	
Farmer	187 (55.33%)	78 (6.32%)	
Student	23 (6.80%)	39 (3.16%)	
Teacher	1 (0.30%)	22 (1.78%)	
Others	13 (3.55%)	100 (8.10%)	
**Month, number (%)**	<0.001
7	10 (2.38%)	16 (1.30%)	
8	69 (16.43%)	400 (32.41%)	
9	232 (55.24%)	736 (59.64%)	
10	89 (21.19%)	81 (6.56%)	
11	20 (4.76%)	1 (0.08%)	

*SD, Standard deviation*.

**Table 2:** Characteristics of Dengue outbreaks in Zhejiang Province from 2004 to 2018.

**Year**	**Site**	**Date**		**Onset date**		**Duration (days)**	**Serotype**	**LC**
		**of report**	**of confirmation**	**of the first case**	**of the last case**			
2004	Cixi, NB	Oct 3	Oct 5	Aug 26	Oct 14	49	1	SA
2009	Yiwu, JH	Sep 2	Sep 8	Jul 20	Oct 4	94	3	SA
2015	Shaoxing	Sep 8	Sep 8	Jul 15	Sep 24	71	2	SA
2016	Huangyan, TZ	Oct 30	Oct 30	Sep 1	Nov 11	71	1	SA
2017	HZ	Aug 23	Aug 23	July 15	Nov 2	110	2	UA
2017	Wenling, TZ	Sep 19	Sep 19	Sep 14	Sep 21	7	1	SA
2017	Wenling, TZ	Oct 10	Oct 10	Oct 3	Oct 13	10	1	SA
2018	Yuhang, HZ	Sep 27	Sep 27	Sep 18	Oct 5	17	1	SA
2018	Shangcheng, HZ	Jul 23	Jul 23	Jul 19	Aug 18	30	1	UA
2018	Jianggan, HZ	Aug 9	Aug 9	Aug 7	Aug 10	3	1	UA
2018	Haishu, NB	Aug 3	Aug 3	Jul 30	Sep 19	78	1	UA
2018	Jiangbei, NB	Aug 7	Aug 7	Aug 1	Sep 21	51	1	UA

*NB, Ningbo; JH, Jinhua; TZ, Taizhou; HZ, Hangzhou; LC, Location; SA, Suburban area; UA, Urban area*.

### Spatial Distribution

**Figure 2:**
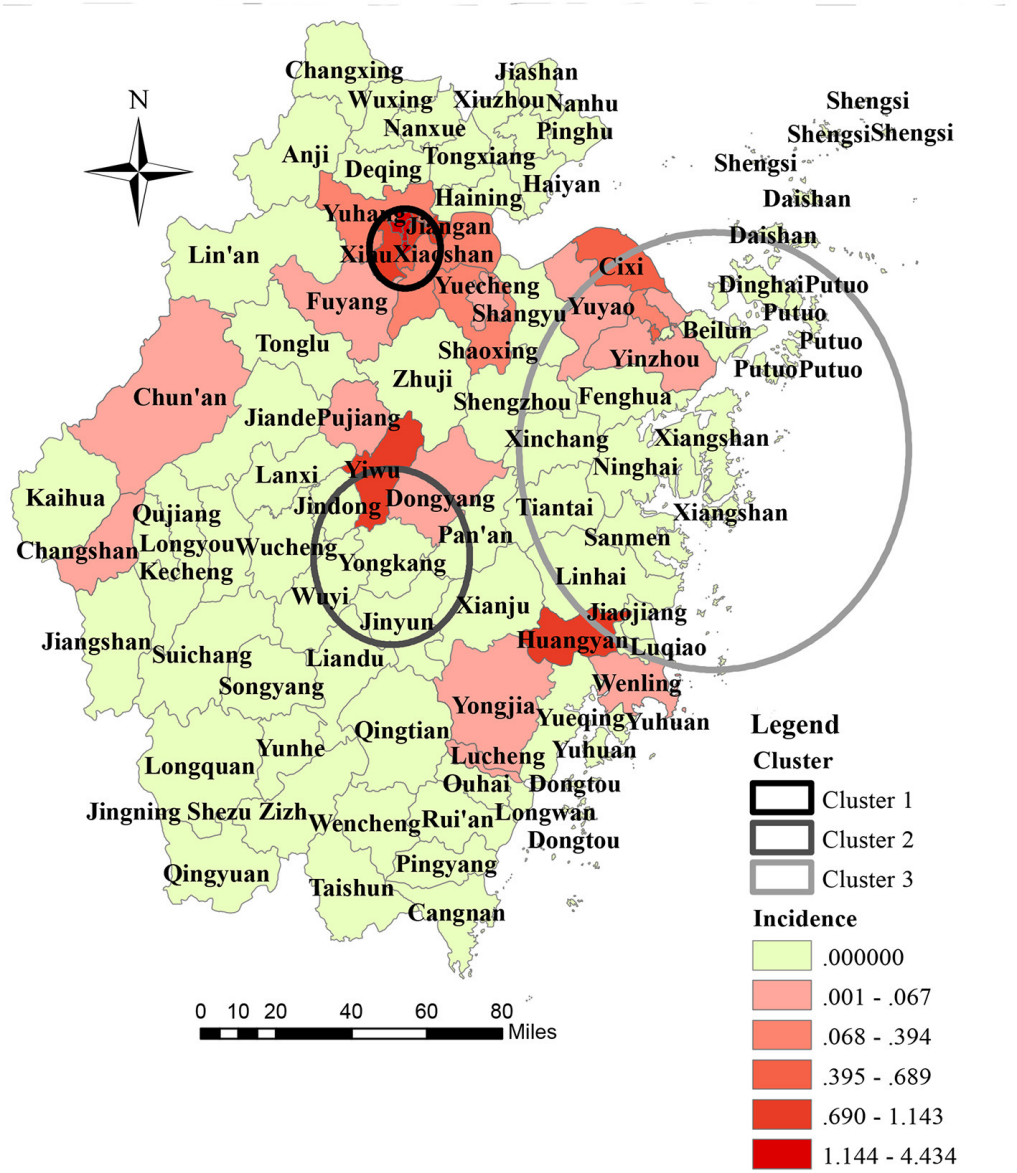
Geographic distribution and space-time cluster of indigenous dengue case in Zhejiang Province from 2004 to 2018.

Seven out of the 11 prefecture cities in ZP reported autochthonous transmission of DENV, and five out of seven prefecture cities confirmed dengue outbreaks during the study period. Hangzhou reported the highest annual mean incidence (9.48 per million), followed by Jinhua (2.56 per million), and Ningbo (1.31 per million). At the prefecture city-level, the three highest annual incidences were reported in Hangzhou in 2017 (123.75 per million), Jinhua in 2009 (38.74 per million), and Taizhou in 2016 (14.55 per million), respectively. At the district-level, 27 out of 93 counties in ZP reported an indigenous dengue case. The highest annual mean incidence was reported in Gongshu (44.34 per million), Shangcheng (37.02 per million), and Xiacheng (36.35 per million) (Figure 2). Other than the outbreak in Hangzhou in 2017 and Shaoxing in 2015, the number of affected counties in a single outbreak was one. In the outbreak in Shaoxing in 2015, dengue cases were reported in Keqiao and Yuecheng. In the dengue outbreak in 2017, cases were reported in 12 out of 15 counties of Hangzhou, except for Lin'an, Tonglu, and Dajiangdong.

The geographical distribution of autochthonous transmission of DENV expanded dramatically after 2016 (Figure 1). One primary cluster (cluster 1) and two secondary high-risk clusters (clusters 2 and 3) were scanned (Figure 2). The primary cluster, located in the northern region of ZP, included seven countries and 986 cases from August to September 2017 [Relative Risk (RR) = 1,910.72, LLR = 5,951.12, *P* < 0.001]. Cluster 2, located in the central region of ZP, included four countries and 185 cases from August to September 2009 (RR = 251.82, LLR = 827.45, *P* < 0.001). Cluster 3, located in the central and east coastal regions of ZP, covered 19 counties and 80 cases from September to October 2004 (RR = 23.30, LLR = 173.49, *P* < 0.001).

### Demographic Characteristics

**Figure 3:**
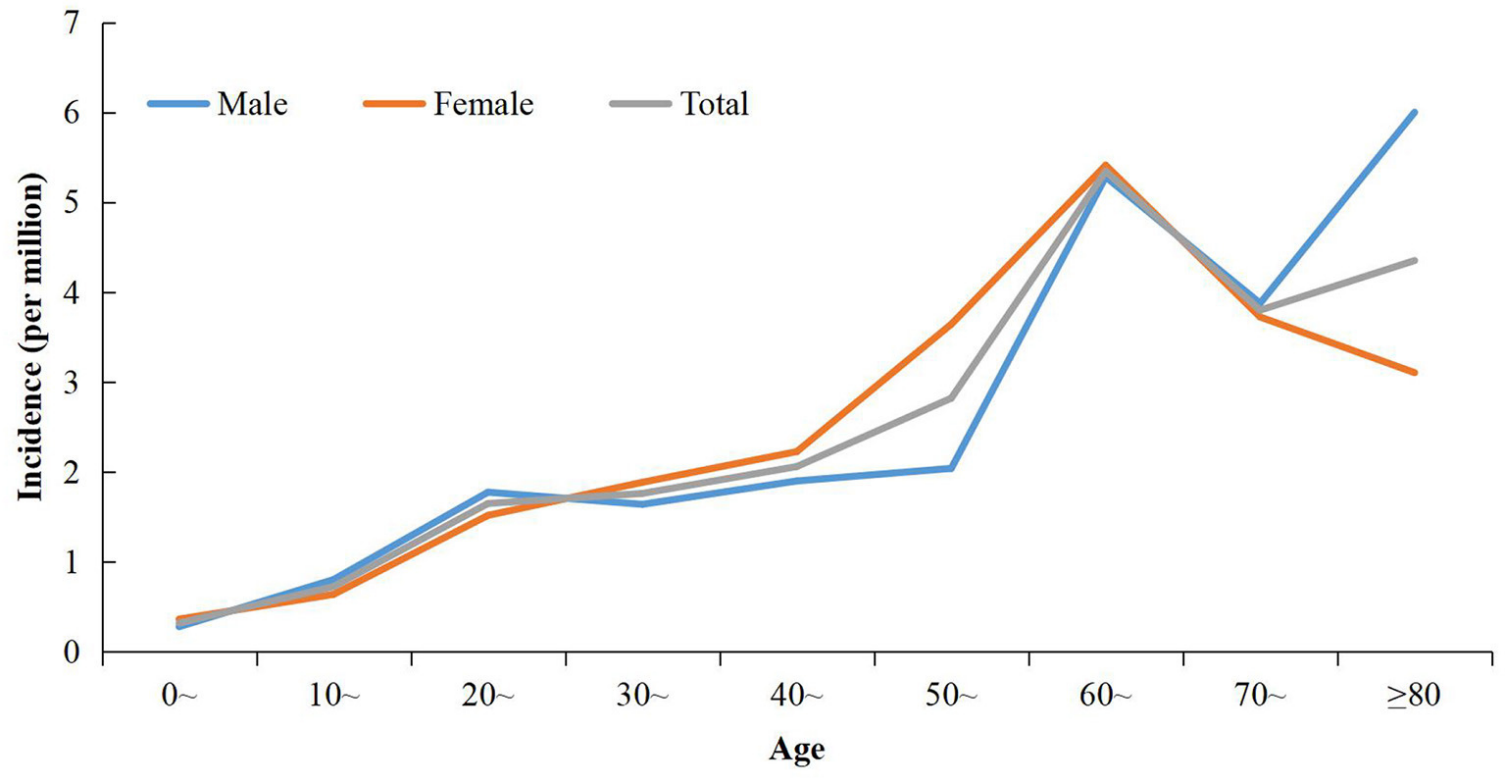
Age-specific incidence of indigenous dengue in Zhejiang Province during 2004–2018.

The overall male:female ratio was 0.93:1. The sex distribution varied significantly in different time periods. The sex ratio in 2004–2016 was 0.71:1, whereas the sex ratio was 1.02:1 in 2017–2018 (χ^2^ = 9.30, *P* = 0.002) (Table 1). The overall incidence in females was higher than that in males during 2004–2018 (2.22 per million vs. 1.97 per million). Although the incidence increased in males and females during 2004–2016 to 2017–2018, the gap between the sexes was reduced greatly. During 2004–2016, the incidence was 0.50 per million for males and 0.74 per million for females. The incidences was 10.80 per million and 11.15 per million, respectively, during 2017–2018. The overall mean age of cases was 48.77 (range, 1–96) years. The age-specific incidence increased steadily with age and peaked for those aged 60–69 years (Figure 3). The highest sex-specific incidence was reported in the group ≥ 80 years for males and 60–69 years for females, respectively (Figure 3). The age distribution was different between the two periods: cases during 2004–2016 were significantly younger than their counterparts during 2017–2018 (*t* = −3.64, *P* < 0.001) (Table 1).

Retiree (23.66%), farmer (16.86%), and worker (12.91%) were the three most common occupations. The distribution of occupation varied significantly between the two periods (χ^2^ = 533.38, *P* < 0.001). Farmer (55.33%), worker (14.20%) and, unemployed or housework (11.54%) were the three most commonly reported occupations during 2004–2016, whereas retiree (29.66%), unemployed or housework (13.78%), and commercial service (13.13%) were the three most commonly reported occupations during 2017–2018 (Table 1).

### Medical Visit and Confirmation

The median onset-visiting time (OVT) was 1 (range, 0–1) day. A significant difference was not observed for cases of different sex, age, from different locations, or year (*P* > 0.05). The median visiting-confirmation time (VCT) was 3 (IQR, 2–5) days. Overall, the VCT of female cases was significantly shorter than that of the male cases (*Z* = −3.069, *P* = 0.002). The stratification analysis indicated that the sex difference was significant during 2017–2018 (*Z* = −2.801, *P* = 0.005) and for urban cases (*Z* = −2.576, *P* = 0.010). The VCT for females was significantly shorter than that of males in cases <60 (*Z* = −2.019, *P* = 0.043) and ≥60 (*Z* = −2.531, *P* = 0.011) years of age. The VCT during 2017–2018 was significantly shorter than that during 2009–2016 (*Z* = −11.009, *P* < 0.001), and the difference remained significant after adjustment for sex, age, and location (*P* < 0.05). Compared with cases live in a non-urban setting, cases living in an urban setting had a higher possibility of early diagnoses (*Z* = −5.558, *P* < 0.001). Considering the limited number of cases living in an urban setting during 2009–2016, a stratified analysis was conducted in cases reported in 2017–2018 to control for potential confounders. Results indicated that cases living in an urban seeing had a longer time interval than those in a non-urban setting during 2017–2018 (*Z* = −2.538, *P* = 0.011). The median onset-confirmation time (OCT) was 4 (IQR, 3–6) days. The OCT was significantly shorter for cases living in an urban setting (*Z* = −10.098, *P* < 0.0001), but the difference was not significant after adjustment for period (*P* > 0.05). The OCT in 2017–2018 was significantly shorter than that in 2009–2016 (*Z* = −11.475, *P* < 0.0001). In total, 65.17% cases visited a clinic or hospital for medical advice at least twice, and 25.68% visited at least thrice.

### Phylogeny of the DENV and Source Tracing

**Figure 4:**
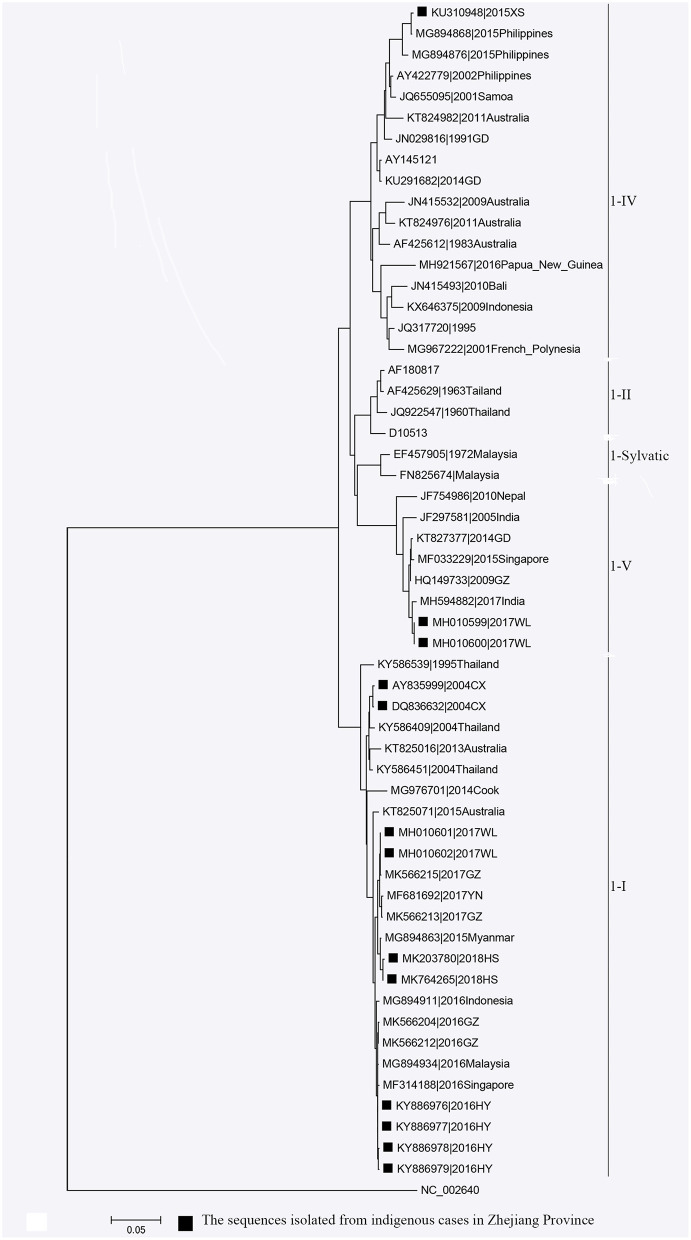
Phylogenetic tree of DENV-1 strains based on the E gene.

**Figure 5:**
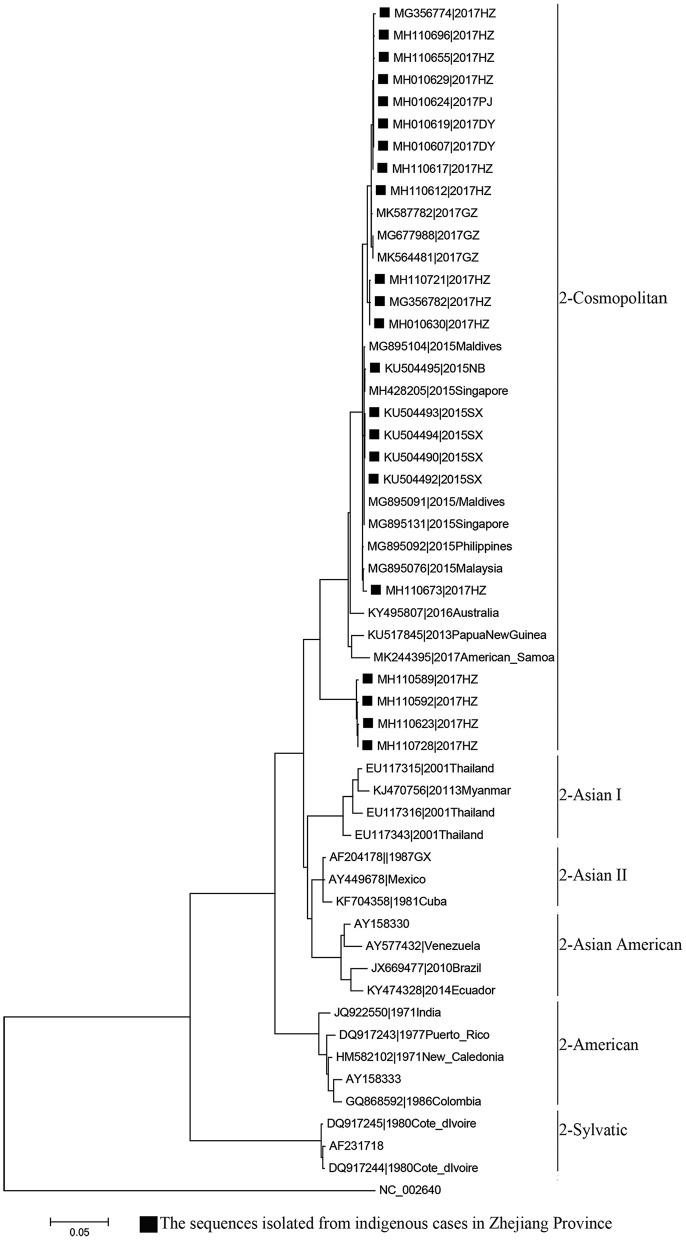
Phylogenetic tree of DENV-2 strains based on the E gene.

**Figure 6:**
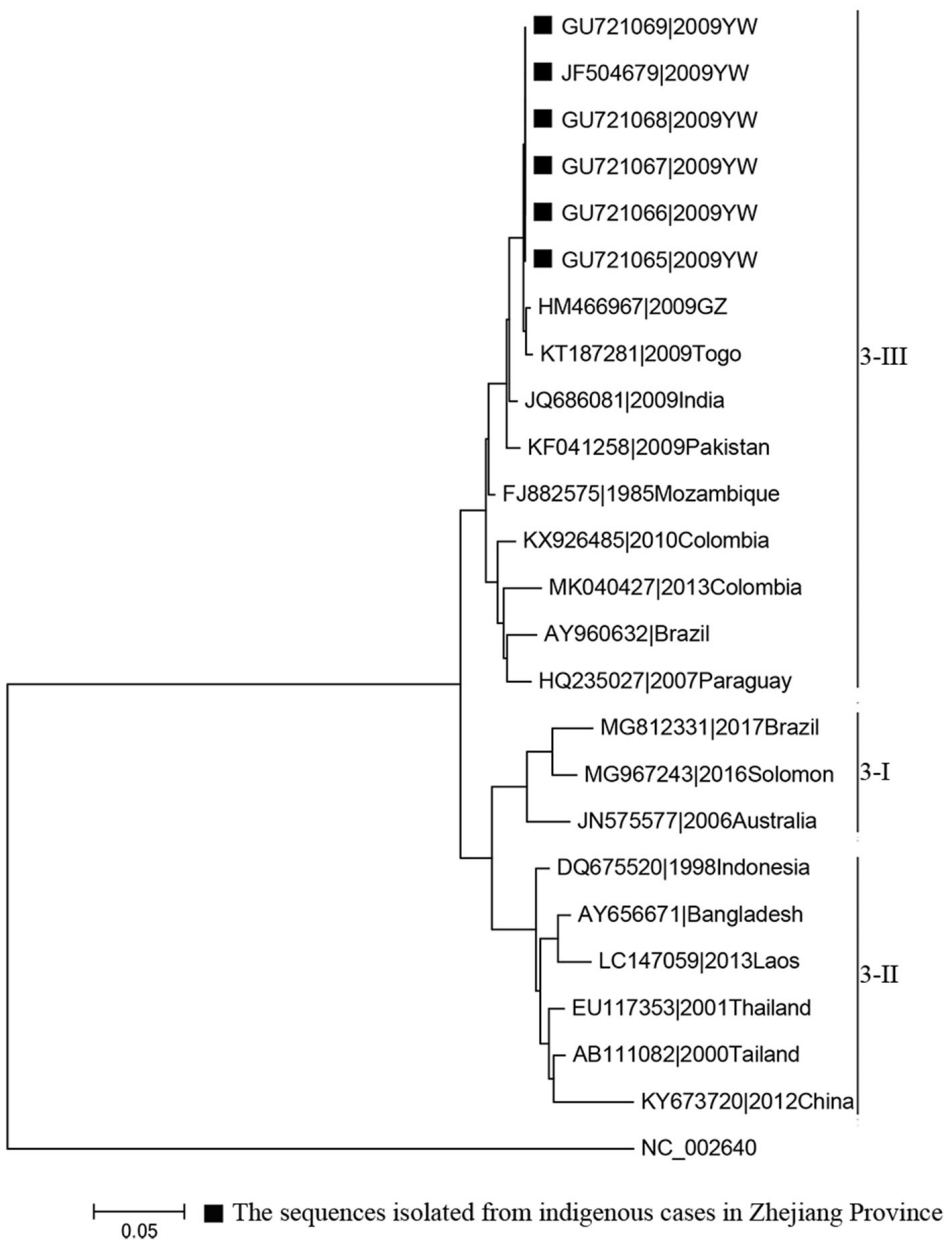
Phylogenetic tree of DENV-3 strains based on the E gene.

Phylogenetic analyses indicated that three serotypes of the DENV (DENV 1–3) were responsible for autochthonous transmission in ZP from 2004 to 2018, with DENV 1 being the most common (Figures 4–6). DENV 1-I was the most frequently reported genotype in DENV 1, and was responsible for autochthonous transmission in Cixi in 2004, Huangyan in 2016, Wenling in October 2017 and Haishu in 2018. DENV1-IV and 1-V were isolated from indigenous cases in Xiaoshan (2015) and Wenling (September, 2017), respectively. DENV 2-cosmopolitan was responsible for autochthonous transmission in Shaoxing (2015), Ningbo (2015), Hangzhou (2017), Pujiang (2017), and Dongyang (2017). The outbreak in Yiwu (2009) was caused by DENV 3-III. Results from BLAST and epidemiological surveys implied that Southeast Asia was the predominant source of infection for autochthonous transmission in ZP (Table 3). The dengue epidemic in Hangzhou in 2017 might have arisen from three sources.

**Table 3:** Source tracing of autochthonous transmission of Dengue in Zhejiang Province.

	Most likely strain identified through BLAST^*^			**Epidemiological evidence**
**Autochthonous transmission^†^**	**Accession number**	**Location (year)**	**Identity (%)**	
Cixi 2004	KY586409	Thailand (2004)	99.06–99.12	A local man returned from Thailand on 18 July 2004. Six days later, he had fever and fatigue. His serum was tested positive for anti-dengue IgG in October.
Yiwu 2009	HM466964	GD (2009)	99.86	_____
	KT187285	Cape Verde (2009)	99.66	
	KT187281	Togo (2009)	99.53	
Shaoxing 2015	MG895131	Singapore (2015)	99.93	_____
	MG895091	Maldives (2015)	99.93	
Ningbo 2015	MG895131	Singapore (2015)	99.93	_____
	MG895091	Maldives (2015)	99.87	
	KU504492	Shaoxing, ZJ (2015)	99.87	
Xiaoshan 2015	MG894868	Philippine (2015)	99.73	Only one male indigenous case was reported. He returned from Philippines with his wife on 23 October. His wife was confirmed as having Dengue on 5 November. The husband developed dengue symptoms on 16 November and tested positive for DENV RNA on 19 November.
Huangyan 2016	MG894928	Malaysia (2016)	99.87–100	_____
	MK566212	GD (2016)	99.86–100	
	MG894926	Thailand (2016)	99.80–99.93	
	MF314188	Singapore (2016)	99.73–99.87	
Wenling 2017(Sep)	MH594882	India (2017)	99.39	The husband of one of the indigenous cases returned from India in the mid-August and his serum tested positive for anti-dengue IgG.
Wenling 2017 (Oct)	MK566227	GD (2017)	100	_____
	MK466341	YN (2017)	99.60	
Hangzhou 2017(a)	MH827547	Malaysia (2017)	99.80–99.93	_____
	MK587782	GD (2017)	99.66–99.93	
Hangzhou 2017(b)	MK564480	GD 2016	99.66–99.80	_____
	MG895168	Thailand 2016	99.66–99.80	
	MH827546	Thailand (2017)	99.53–99.66	
Hangzhou 2017(c)	MG895150	Thailand (2016)	99.53–99.66	_____
	MG895121	Viet Nam (2015)	99.46–99.60	
Dongyang 2017	MH110734	Hangzhou, ZJ (2017)	100	_____
Pujiang 2017	MH110734	Hangzhou, ZJ (2017)	100	_____
Haishu 2018	MG894863	Myanmar (2015)	99.53–99.66	_____
	MG564080	Thailand (2015)	99.39–99.53	

* *The most likely strains were those with the highest percent identity and isolated in the same year or 3 years before autochthonous transmission was identified in Zhejiang Province*.

† *The sequence of autochthonous transmission at Yongjia, and Lucheng of Wenzhou in 2014 was unavailable. The sequence of autochthonous transmission in Zhejiang Province in 2018, except Haishu of Ningbo, was unavailable*.

*BLAST, Basic Local Alignment Search Tool; DENV, dengue virus; GD, Guangdong Province of China; ZJ, Zhejiang Province of China; YN, Yunnan Province of China*.

### Vector Surveillance

**Figure 7:**
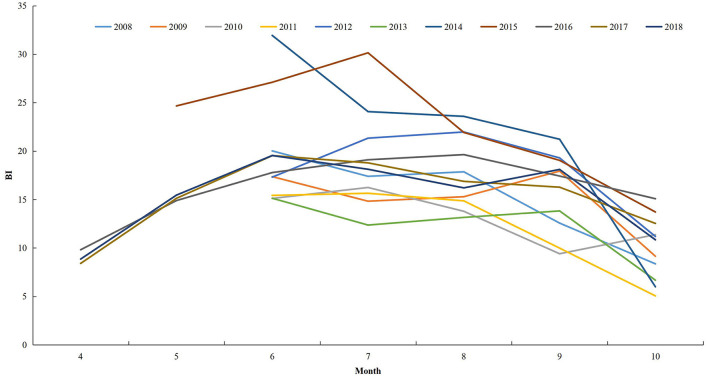
The monthly mean Breteau Index in Zhejiang Province from 2008 to 2018.

In general, the BI in ZP increased from April and peaked in June–July, then decreased (Figure 7). From 2008 to 2018, the monthly mean BI > 5 for June to October. The monthly mean BI > 10 for June–September from 2012 to 2018. The maximum mean BI reported was in July 2015.

## Discussion

Mirroring the trends on the Chinese mainland and worldwide, ZP has witnessed the emergence of dengue in recent years with an increase in frequency, magnitude, and geographic reach. Moreover, the seasonal distribution of autochthonous transmission has changed over time. During 2004–2016, the percentage of cases between September and October was the highest. However, after 2016, August and September had the highest incidence. Three reasons may underlie this change. The first is climate change. Studies have indicated that temperature, precipitation, and humidity to be related to the seasonal variations of the dengue epidemic (21, 22). The second reason is the surge in travel and increase of imported cases. Convenient transportation means more imported cases, which pose a high risk of autochthonous DENV transmission in the mosquito-abundance season (22, 23). The third reason is advancement in the awareness and diagnosibility of dengue. With wide health education and frequent professional training, awareness of dengue has increased among the general public and healthcare professionals. Furthermore, wide use of rapid diagnostic tests has made the diagnosis of dengue more convenient.

Indigenous cases were distributed mainly in the coastal and central parts of ZP. Three significantly high-risk clusters were identified. The studies conducted in dengue non-endemic regions indicated that the core contributors to the spatial heterogeneity of dengue were: urban village; urban-rural fringe zone; dense population, human mobility, gross domestic product per capita; Normalized Difference Vegetation Index; number of imported cases; subway network; density of road networks (22, 24–27). The three high-risk clusters we identified were characterized by a dense population, high human mobility, high incidence of imported cases, dense road networks, and advanced economy. Hangzhou and Ningbo, which were covered by a high-risk cluster, were the only cities equipped with a subway in ZP by 2018. Additionally, five of the seven on-service civilian airports were in the region of high-risk clusters. From 2017, the high-risk area had changed from a suburban area to an urban area. This phenomenon could be attributed (at least in part) to rapid urbanization in ZP. During urbanization, a large suburban area underwent construction (infrastructure construction, housing demolition and relocation) to become new urban area. A village in a suburban area is an “ideal” residence for low-income workers, because of relatively cheap rents and convenient transportation. However, poor sanitation and crowding make residents vulnerable to communicable diseases. Housing demolition/relocation is a policy for better development and logical planning of urban areas. From 2016, this policy was implemented rapidly in most parts of China (including ZP) because of sustainable and high-speed domestic economic development. A village in a suburban area is one of the main targets of this program. To some extent, it influenced the shift of high-risk DENV transmission in ZP.

As a whole, more indigenous cases were female in ZP. The sex distribution varied across age. Sexual inequality was less noticeable in cases during 2017–2018. The sex-based distribution of dengue was not consistent in studies from different regions. Female predominance has also been noted previously (28, 29). However, some studies in dengue-endemic Southeast Asian countries have indicated males to be more susceptible to DENV infection (30, 31). The difference in dengue predominance among the sexes for populations in different regions and for different ages might be related to the different risks of exposure, dengue precautions taken, health-seeking behavior, and disease severity after infection (32–35).

In ZP, the incidence of dengue increased with age; this result is consistent with data from studies in other non-endemic regions (36). However, in dengue-endemic countries, dengue affects the young disproportionally, especially those aged ≤15 years (37, 38). This disparity in age distribution between non-endemic and endemic regions is related to the difference in the risk and frequency of DENV exposure and immunity of the population. In endemic areas, due to perennial or periodic dengue epidemics, most of people become infected in childhood (and sometimes prenatally). With continuous exposure, the seroprevalence of dengue antibodies increases with age in endemic areas, making younger people more vulnerable (39, 40). In non-endemic areas, the seroprevalence is significantly lower and age-independent in the context of the occasional and transitory transmissions, and people from all age groups are susceptible to DENV infection (41). In our study, the cases reported in 2004–2016 were significantly younger and more frequently reported to be farmers compared with those reported during 2017–2018. This disparity could be attributed to differences in demographics. As mentioned above, all outbreaks during 2004–2016 took place in suburban areas. The latter contain ideally rental places for peasant-workers, who are usually young and energetic. Outbreaks during 2017–2018 occurred mainly in old urban neighborhoods, where the proportion of retirees was slightly higher.

Our investigation on medical visits indicated that almost all cases would ask for medical advice if symptoms appeared, and repeat visits were common. The median VCT and OCT was 3 (IQR, 2–5) days and 4 (IQR, 3–6) days, respectively. These data indicated that the delay in the diagnosis could be attributed to healthcare providers' unfamiliarity with dengue and the atypical symptoms of the disease. The OCT of indigenous cases was similar with that reported for the whole of China in 2016 (42). The VCT and OCT in ZP had reduced significantly in recent years, and this trend has also been noted in China and Australia (43, 44). The VCT for females was shorter than that for males. This phenomenon might be related to: (i) females being more concerned about their physical health and better at communication; (ii) sex-based differences in disease development (45). The critical phase, usually 4–6 days after illness onset, is characterized with fever clearance and development of severe manifestations in a small number of cases (46). Closer monitoring is necessary in this phase to reduce the risk of death. Our study indicated that ≥20% cases in ZP would not be diagnosed accurately as dengue in the critical phase.

DENV 1 was the most common DENV strain responsible for DENV transmission in ZP, which was in accordance with that noted in the whole of China (47). Moreover, the annual distribution of the serotype in ZP was consistent with that in Guangzhou during 2009–2016 (48). BLAST results indicated that Southeast Asia was the main source of infection for autochthonous transmission in ZP, and it was also the main source for imported cases in ZP and other dengue non-endemic countries in Asia and Europe (49, 50). All four serotypes of DENV were co-circulating in Southeast Asia and were imported to China by travelers. DENV 1 (followed by DENV 2) was the most common strain responsible for autochthonous transmission in ZP and Guangdong Province in China (51, 52). This disproportional distribution in serotype may be due to four main reasons. The first is the serotype-specific difference in clinical manifestation (53). The second is the serotype-specific difference in competency of *A. aegypti* and *A. Albopictus* (54, 55). *A. albopictus* is the only vector responsible for DENV transmission in ZP, whereas *A. aegypti* is the main vector (followed by *A. albopictus*) in Southeast Asia. The third reason is the difference in compatibility and fitness of the DENV in different regions (56, 57). The forth reason is the serotype-specific difference in susceptibility for different ethnicities (55).

To stop further spread of Dengue, an emergency program to control mosquitoes should be initiated by local government after a dengue case has been reported during April to November in ZP. This program would involve killing larvae and adult mosquitoes with insecticides, removing all standing water and potential mosquito habitats, and health education on mosquito-control practices. A routine mosquito-control program should also be launched according to the risk assessment. A routine mosquito-control program would mainly involve removal of mosquito (and the potential mosquito) habitats and health education. Insecticides should also be used if necessary. A routine mosquito-control program should be conducted before 15 May if autochthonous transmission of DENV is reported in the previous year.

Our study had two main limitations. First, the capability of early detection and diagnosis varied across the study period, which is a potential confounder and was not tackled. Second, the capability and intensity of case-searching after the verification of autochthonous transmission was uneven across ZP, and might have confounded the geographic distribution.

## Conclusion

In recent decades, ZP experienced an increase in incidence, frequency, and geographic reach of Dengue. Four main changes were noted during this period: (i) earlier seasonal peak; (ii) transformation of high-risk areas from suburban to urban; (iii) higher proportion of older cases; (iv) higher proportion of cases whose occupations were retiree, worker, cadre or clerk. Further research is needed to explore the driver behind these changes. Southeast Asia was the predominant source of infection, and DENV 1 and DENV 2 were the most frequently reported serotypes. After years of effort, the diagnosibility of dengue improved. Nevertheless, a gap exists and further improvement is needed in consideration of the increasing risk of autochthonous transmission and potential for severe dengue attributed to secondary infection.

## Data Availability Statement

The raw data supporting the conclusions of this article will be made available by the authors, without undue reservation.

## Ethics Statement

Ethical Approval was obtained from the Chinese CDC Ethics Committee (No. 201214). Written informed consent from the participants' legal guardian/next of kin was not required to participate in this study in accordance with the national legislation and the institutional requirements.

## Author Contributions

JR, ZW, and JS conceptualized the research. ZC, FL, and EC managed the overall project. HY, ZG, YL, CL, XS, RZ, SG, JR, and JS were responsible for the epidemiological survey. YH was responsible for the laboratory testing. JR and JS were responsible for data analyses. JR, HY, and ZM were responsible for writing the first draft of the manuscript. The authors approved the final version of the manuscript for submission.

## Funding

This work was funded by the medical research program of Zhejiang Province (2019ZD003, 2020KY092, 2021KY622, and LGF20H260001), State Project for Scientific & Technological Development of the 13th Five-Year Plan (2017ZX10303404003006, 2017ZX10303404005004, and 2017ZX10303404008002), National Basic Research Program of China (973 Program) (2012CB955504), and National Natural Science Foundation of China (81273139, 81602898, and 81872675).

## Conflict of Interest

The authors declare that the research was conducted in the absence of any commercial or financial relationships that could be construed as a potential conflict of interest.

## Publisher's Note

All claims expressed in this article are solely those of the authors and do not necessarily represent those of their affiliated organizations, or those of the publisher, the editors and the reviewers. Any product that may be evaluated in this article, or claim that may be made by its manufacturer, is not guaranteed or endorsed by the publisher.
